# Does peri-implant bone loss affect the LL-37 and proteinase 3 levels in peri-implant sulcus fluid?

**DOI:** 10.1186/s40729-020-00240-8

**Published:** 2020-08-04

**Authors:** Oya Turkoglu, Candan Efeoglu, Harika Atmaca

**Affiliations:** 1 Department of Periodontology, School of Dentistry, Ege University, Bornova, 35100 Izmir, Turkey; 2 Department of Oral Surgery, School of Dentistry, Ege University, Izmir, Turkey; 3 Department of Biology, Celal Bayar University, Manisa, Turkey

**Keywords:** Cathelicidin LL-37, Peri-implantitis, Peri-implant sulcus fluid, Proteinase 3

## Abstract

**Background:**

Inactive human cathelicidin antimicrobial peptide is present in neutrophils, and proteinase 3 activates this peptide by producing active LL-37 peptide. LL-37 acts as a defensive peptide in the oral tissues. In the present study, the aim was to evaluate LL-37 and proteinase 3 levels in peri-implant sulcus fluid (PISF) in implants with and without peri-implantitis.

**Methods:**

Patients who simultaneously had dental implants with peri-implantitis and without peri-implantitis were included in the study. Forty-four samples with peri-implantitis and 34 samples without peri-implantitis from 16 patients were obtained. Intraoral evaluations such as pocket depth, modified sulcus bleeding index, and modified plaque index were noted. Enzyme-linked immunosorbent assay was used for the evaluation of PISF LL-37 and proteinase 3 levels.

**Results:**

PISF volume was significantly increased in the implants with peri-implantitis than those without peri-implantitis (*p* < 0.05). No difference was present between PISF LL-37 and proteinase 3 total amounts between the implants with and without peri-implantitis (*p* > 0.05). Pocket depths and PISF LL-37 and proteinase 3 levels were not correlated in the groups (*p* > 0.05).

**Conclusions:**

PISF volume might be increased in response to peri-implant bone destruction. However, peri-implant tissue destruction caused by peri-implantitis does not seem to affect PISF LL-37 and proteinase 3 levels.

## Introduction

Peri-implantitis is a disease affecting tissues around functional dental implants and an inflammatory condition of mucosa that is accompanied by loss of surrounding bone of the implant [1]. Dental implants are a more frequently preferred alternative for reconstruction of edentulous ridge with artificial teeth, and this inevitably leads to a higher incidence of diseases affecting the tissues around the implant. Finally, the disease might lead to implant loss. Zitzmann et al. stated that peri-implantitis affects 28 and 56% of the adults and 12 and 43% of the implants [1].

Peri-implant sulcus fluid (PISF) is similar to gingival crevicular fluid as it reflects the inflammatory response [2, 3]. It has been stated that the exudate leaking from the peri-implant tissue into the peri-implant sulcus increases during the inflammatory process [4]. Up to date, various cytokines in peri-implant sulcus fluid (PISF) have been investigated in peri-implant disease [4–7]. Matrix metalloproteinase-8 levels were found to be higher in peri-implantitis sites [5]. Similarly, researchers have demonstrated that interleukins 1β, 6, and 10 and TNF-α concentrations were related to inflammatory response during peri-implantitis [6]. Acipinar et al. has demonstrated that FGF-23 and vitamin D seem to affect peri-implant bone health [7]. Previously, it was shown that peri-implantitis did not give rise to an increase in osteocalcin, osteopontin, and osteonectin levels in PISF [4]. Hence, studies investigating the biomarkers that reflect the changes during peri-implant inflammation continue.

Antimicrobial peptides in the oral tissues are crucial participants of the equilibrium between healthy and diseased states [8, 9]. Human cathelicidin antimicrobial peptide has a cathelin-like domain and a LL-37 part [8]. This antimicrobial peptide is expressed especially by neutrophils and epithelial cells [10, 11]. Inactive human cathelicidin antimicrobial peptide is present in the neutrophil granules [8, 12]. Following neutrophil stimulation, proteinase 3 activates this inactive peptide to produce cathelin-like domain and active LL-37 [12]. Proteinase 3 degrades extracellular matrix proteins, activates platelets, and induces the apoptosis [13–15]. Both LL-37 and proteinase 3 have effect on the nonoxidative killing of microorganisms [16, 17].

Gingival crevicular fluid (GCF) LL-37 and proteinase 3 levels were investigated in the presence of different periodontal diseases [18–24]. Researchers demonstrated that LL-37 antimicrobial peptide is related to periodontal inflammation [18, 21–24]. It was denoted that increased GCF LL-37 amounts in periodontal tissue destruction might be related to the innate immunity in inflammation of periodontal tissues [18]. Laugisch et al. showed that GCF proteinase 3 activity was the highest in gingivitis patients [20]. Our previous study revealed that proteinase 3 levels in GCF was higher in periodontally diseased patients compared to those in periodontally healthy, however similar between those of the periodontally diseased [21]. Probing depths might not be measured effectively because of the inappropriate prosthetic restorations. Additionally, it is known that the macro design of the implant and surface features could prevent appropriate probing and periodontal probe penetration into the pocket [25]. Since standard clinical measurements including pocket depth and bleeding upon probing are of limited diagnostic value for peri-implant diseases, additional prognostic criteria would be invaluable for detecting peri-implant diseases. Therefore, in this study, the hypothesis is that peri-implant tissue destruction caused by peri-implantitis might affect the LL-37 and proteinase 3 amounts in peri-implant sulcus fluid (PISF). Accordingly, the aim is to evaluate LL-37 and proteinase 3 amounts in PISF in implant sites with and sites without peri-implantitis, in order to detect temporal changes regarding the progression of the disease.

## Materials and methods

Patients having implants with peri-implantitis and without peri-implantitis simultaneously were identified to be included into study. A total of 16 patients who had both peri-implantitis and non-peri-implantitis sites were included into study. Each of the 16 patients had both study groups: peri-implantitis and non-peri-implantitis. Forty-four implants with peri-implantitis and 34 implants without peri-implantitis from 16 patients were evaluated. Participants were selected among the individuals who admitted to the Periodontology Department, School of Dentistry, Ege University. All participants gave informed consents. Research protocol was approved by the Ege University Ethics Committee. Patients who had undertaken antibiotic in the past 3 months, who have infectious diseases, systemic diseases, and immunological disorders; women who were pregnant or who breastfeed; and smokers were excluded. Implants included in the study were minimum placed a year previously. They were also loaded for at least 6 months in a functional way.

### Intraoral measurements

Intraoral measurements including pocket depth, attachment loss, bleeding upon probing, and plaque index were measured in the whole mouth to determine the periodontal condition of the patient [26, 27]. Pocket depth was measured using a plastic periodontal probe. Modified sulcus bleeding index (MSBI), modified plaque index (MPI), and suppuration in the implants were evaluated [28]. Bone level around the implant was inspected by a peri-apical radiograph. A calibrated periodontist carried out all periodontal and peri-implant measurements.

### Study groups

#### Peri-implantitis group

If an implant has bone loss more than 2 mm from the implant platform except for normal bone remodeling and pocket depth deeper than 4 mm and bleeding upon probing, it was diagnosed as peri-implantitis [29].

#### Non-peri-implantitis group

If an implant with or without bleeding upon probing has no radiographic bone loss except normal bone remodeling, it was diagnosed as non-peri-implantitis.

### Collecting PISF samples

PISF sampling was performed 1 day after the clinical examination. PISF samples were obtained from implants with and without peri-implantitis on the same patient. Three or five PISF samples (samples with or without peri-implantitis) were obtained from each patient. A total of 78 samples (44 samples with peri-implantitis and 34 samples without peri-implantitis) were provided from 16 patients. PISF sampling was performed at interproximal surfaces of the implants as described before [4].

### Laboratory analysis

Paper strips were incubated in 200 μl 0.5% Tween solution with 0.1 M phosphate-buffered saline for 15 min. Then enzyme-linked immunosorbent kits^1^^,^^2^ were used for determining PISF LL-37 and proteinase 3 levels according to the directives of the kit. According to the kits, minimum detectable limits for LL-37 was 0.395 ng/ml. Minimum detectable limits for proteinase 3 was 0.236 ng/ml. While making a calculation of the levels of the markers investigated, dilution rates were taken into consideration and results were presented as total amounts.

### Statistical analysis

In order to achieve 95% power, at a medium effect size of 0.5 and with a significance level *α* = 0.05 using a two-sided paired *t* test, a minimum sample size was estimated as 54 (G*Power version 3.1.9.2, Franz, Universitat Kiel, Germany). Categorical data including MPI and MSBI were defined using observed frequencies and percentages. Continuous variables including pocket depth, PISF volume, and PISF LL-37 and proteinase 3 were given by the means and standard deviations with the statistical package^3^. Since the number of implants for each group and the number of implants for each person were unequal, the study design was unbalanced. Accordingly, we included random person effect and fixed effect for group in the model when comparing pocket depth, PISF volume, and PISF LL-37 and proteinase levels between two groups, and this model was analyzed by using SAS 9.3^4^. Group, jaw, and interaction effect between group and jaw were also evaluated for PISF LL-37 and proteinase 3 levels. Pearson analysis was applied for assessing the correlation between pocket depth and PISF volume and PISF LL-37 and proteinase 3 levels.

## Results

### Demographics and intraoral data

Demographic evaluation of 16 patients was performed. The mean age of the study participants was 54.06 years (42–66 years). Eight patients of the study participants were female. Four out of 16 patients were periodontally healthy, 4 of them had gingivitis, and 8 had chronic periodontitis [30]. Nineteen of the 44 peri-implantitis sites in 16 patients were located on the maxilla, and 25 were located on the mandibula. Nineteen of the 34 non-peri-implantitis sites in 16 patients were on the maxilla, and 15 were on the mandibula. All participants were non-smokers. The mean time between delivery of the prosthesis and the appointment of examination were 8 ± 2 years (mean ± standard deviation).

Peri-implant data of the sampling sites is demonstrated in Table 1. Pocket depths of the implants with peri-implantitis was significantly increased in comparison to those without peri-implantitis (*p* < 0.0001). MSBI and MPI were significantly increased in implants with peri-implantitis in comparison to implants without peri-implantitis (*p* < 0.05). PISF volume was increased in implants with peri-implantitis compared to implants without peri-implantitis (*p* < 0.05).

**Table 1:** Intraoral findings of sampling sites

Intraoral parameters (mean ± SD or med (min–max))	Peri-implantitis (*N* = 44)	Non-peri-implantitis (*N* = 34)
Pocket depth	6.5 ± 1.4^*^	4 ± 1.3
MPI	2.5 (2–3)^*^	2 (0–3)
MSBI	2 (2–3)^*^	1 (0–2)
PISF	0.62 (0.14–0.99)^*^	0.33 (0.11–0.75)

*MPI* modified plaque index, *MSBI* modified sulcus bleeding index, *PISF* peri-implant sulcus fluid

^*^Significant difference from non-periodontitis group

### PISF LL-37 and proteinase 3 levels

**Fig. 1:**
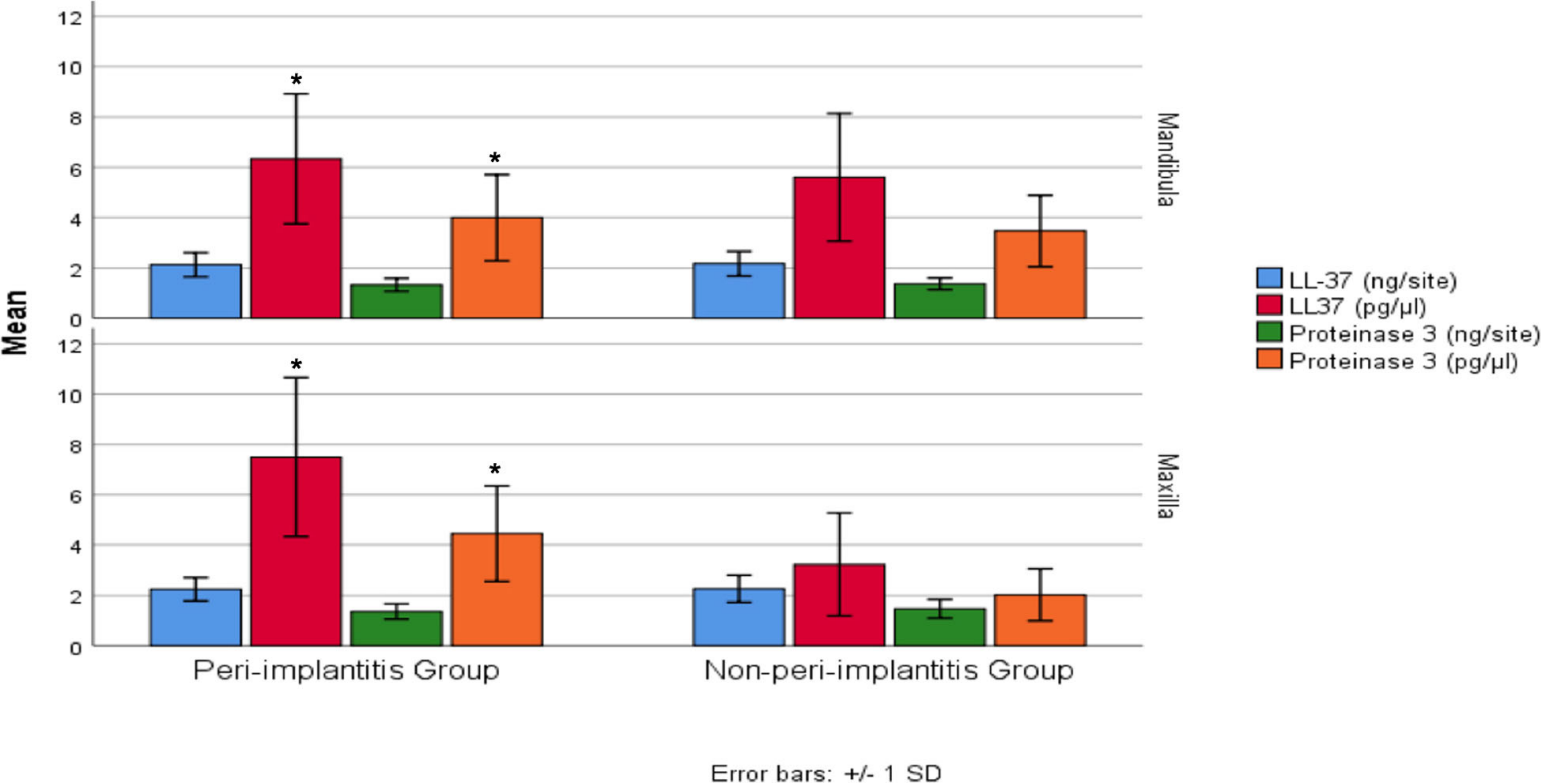
PISF LL-37 and proteinase 3 levels in the study groups (mean ± SD). ^*^Significant interaction between group and jaws

Figure 1 shows PISF LL-37 and proteinase 3 levels in implant sites with and without peri-implantitis. No significant difference was observed in PISF LL-37 total amounts between implant sites with and without peri-implantitis (*p* = 0.585). There was no significant difference in PISF proteinase 3 total amounts between peri-implantitis sites and non-peri-implantitis sites (*p* = 0.083). As to concentrations, there was a significant interaction in PISF LL-37 level between disease and jaw (*p* < 0.05). Similarly, significant interaction was observed in PISF proteinase 3 concentrations (*p* < 0.05). Correlations between PD and PISF LL-37 and proteinase 3 levels are presented in Table 2. There was no significant correlation between sampling sites’ pocket depths and LL-37 total amounts (*p* = 0.506, *r* = 0.077). Sampling sites’ pocket depths and proteinase 3 total amounts were not correlated either (*p* = 0.963, *r* = − 0.005). However, there were significant correlations between pocket depth, PISF LL-37 concentration (*p* = 0.000, *r* = 0.461), and proteinase 3 concentrations (*p* = 0.000, *r* = 0.478). As expected, pocket depth values of sampling sites were correlated with PISF volume (*p* = 0.000, *r* = 0.496).

**Table 2:** Correlations between PD of sampling site and PISF LL-37 and proteinase 3 levels

Periodontal parameter		LL-37 (ng/site)	LL-37 (pg/μl)	Proteinase 3 (ng/site)	Proteinase 3 (pg/μl)	PISF(μl)
PD	*r*	0.077	0.461	− 0.005	0.478	0.496
	*p*	0.506	0.000	0.963	0.000	0.000

*PD* Probing depth, *PISF* peri-implant sulcus fluid

## Discussion

It is stated that the assessment of proinflammatory activity in saliva and GCF could give information about the progress of the periodontal and peri-implant diseases [31–34]. Up to date, proinflammatory cytokines and antimicrobial peptides in GCF and saliva are investigated in various periodontal diseases [18, 24, 31, 35]. Although proinflammatory cytokines, matrix metalloproteinases, and proteolytic enzymes are well studied [21, 35–37], there is no study investigating antimicrobial peptides in peri-implant diseases. As far as we know, PISF LL-37 antimicrobial peptide and its activator-proteinase 3 levels were investigated in peri-implant diseases for the first time with this research. Our findings demonstrated no important changes in PISF LL-37 and proteinase 3 total amounts between implants with and without peri-implantitis. Additionally, total amounts of PISF LL-37, proteinase 3, and pocket depth of sampling sites were not correlated.

It has been demonstrated that GCF reflects the inflammatory state that based on clinical status of the periodontal tissues [38]. In the literature, it has been stated that total amounts per standard sampling time is a better indicator of relative GCF constituent activity rather than GCF concentrations [39–41]. Besides, it has been suggested that PISF is similar to GCF as it reflects the inflammatory response [5]. Therefore, in the present study, PISF LL-37 and proteinase 3 levels were reported as total amounts per time of collection instead of reporting their concentration.

PISF volume of implants with peri-implantitis was increased in comparison to sites without peri-implantitis in the current study, and this result is in consistent with the other researches [42, 43]. Since PISF is an inflammatory exudate, one can reasonably expect to have elevated PISF volume in the presence of peri-implant tissue destruction. Epithelial cells and neutrophils express LL-37 antimicrobial peptide. In the presence of inflammation, blood vessels dilate and neutrophils migrate from the vessels to inflammation area. Studies demonstrated that GCF samples from periodontitis and gingivitis patients had increased levels of LL-37 [18, 21–24]. Our results showed that although PISF volume in implants with peri-implantitis was increased, PISF LL-37 levels in sites with and without peri-implantitis were similar. This could be a result of the compromised blood circulation around dental implants. The vascular supply to the zone of supra-alveolar connective tissue comes from the capillaries of the connective tissue under the epithelium, the vascular plexus near the junctional epithelium and the periodontal ligament [44]. Nevertheless, the vascular supply of peri-implant mucosa comes from the supra-periosteal vessels of the alveolar ridge only [44]. Peri-implant mucosa is lack of the vascular supply of the periodontal ligament since there is no periodontal ligament between the implant and the surrounding bone.

Polymorphonuclear neutrophil granules have proteinase 3 which is expressed following its stimulation [44]. Several studies exhibited that GCF proteinase 3 levels were elevated in sites with periodontal tissue destruction in comparison to healthy periodontal tissues [19–21]. Similarly, in our previous study, it was presented that proteinase 3 levels in GCF were increased in patients with periodontal tissue loss in comparison to healthy controls, but similar between periodontitis and gingivitis subjects [21]. In this current study, proteinase 3 levels in PISF were similar between peri-implantitis and non-peri-implantitis sites. Reduced blood circulation of the supra-alveolar connective tissue of dental implants compared to natural dentition might lead to a relative reduction in the number of neutrophils migrating from peri-implant connective tissue to peri-implant sulcus. This in turn might be the reason for similar proteinase 3 levels in PISF between the groups. Additionally, the presence of sites with peri-implant mucositis as well as healthy peri-implant tissues in non-peri-implantitis group might contribute to the above mentioned finding in the present study. Therefore, studies investigating PISF proteinase 3 levels in peri-implant healthy, peri-implant mucositis, and peri-implantitis might yield more information regarding the pathogenesis of peri-implant diseases. Both proteinase 3 and LL-37 levels in PISF were similar in study groups in this current study. This finding is not unexpected considering the fact that proteinase 3 is the enzyme cleaving hCAP18 to mature LL-37 peptide, and both proteinase 3 and LL-37 are mainly expressed by neutrophils. Therefore, similar levels of these two molecules in PISF is to be anticipated.

There are some limitations in the present study. PISF samples were obtained from implants with prosthetic restorations that make the sample collection difficult. Second, although the control group in this current study comprised of implants without peri-implantitis, some of these implants had peri-implant mucositis. Since the purpose of this current study was to evaluate PISF proteinase 3 and LL-37 levels in the presence of peri-implant tissue destruction, implants with peri-implant mucositis were also included in the control group. Although implants having peri-implant mucositis did not have peri-implant tissue destruction, they had mucosal inflammation. Therefore, studies evaluating the influence of mucosal inflammation on the PISF proteinase 3 and LL-37 levels would provide detailed information regarding the levels of these molecules during the inflammatory process of peri-implant diseases.

## Conclusion

Peri-implantitis might increase PISF volume in response to peri-implant bone loss. However, peri-implant tissue destruction caused by peri-implantitis does not seem to affect PISF LL-37 and proteinase 3 levels. Within the limits of this current study, we concluded that PISF LL-37 and proteinase 3 levels could not be used as prognostic criteria for detecting peri-implant bone loss.

## Data Availability

The datasets used and/or analyzed during the current study are available from the corresponding author on reasonable request.

## Abbreviations

GCF: Gingival crevicular fluid
PISF: Peri-implant sulcus fluid
MSBI: Modified sulcus bleeding index
MPI: Modified plaque index

## Footnotes

1 Human LL-37 Enzyme-linked immunosorbent kit, Mybiosource

2 Human Proteinase 3 Enzyme-linked immunosorbent kit, Mybiosource

3 SPSS Inc., version 21.0, Chicago, IL, USA

4 SAS Institute Inc., Cary, NC, USA, PROC MIXED
